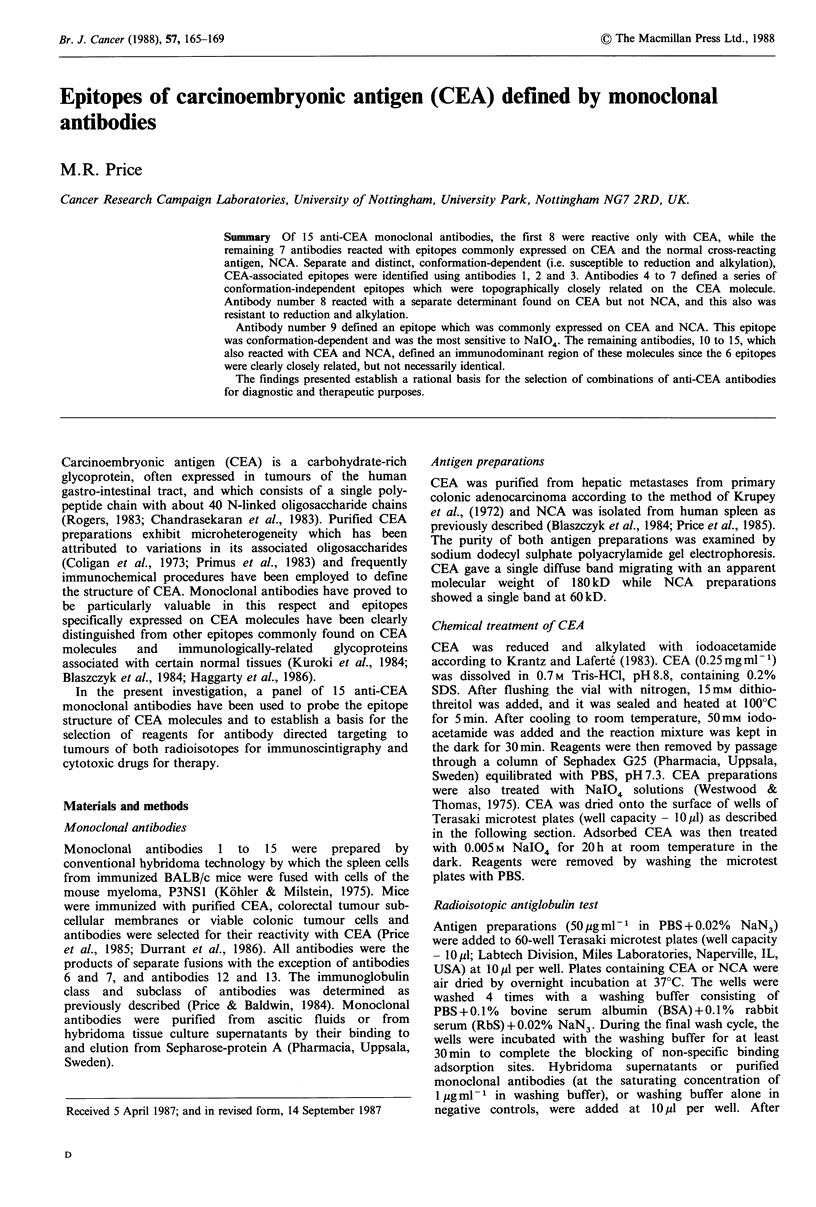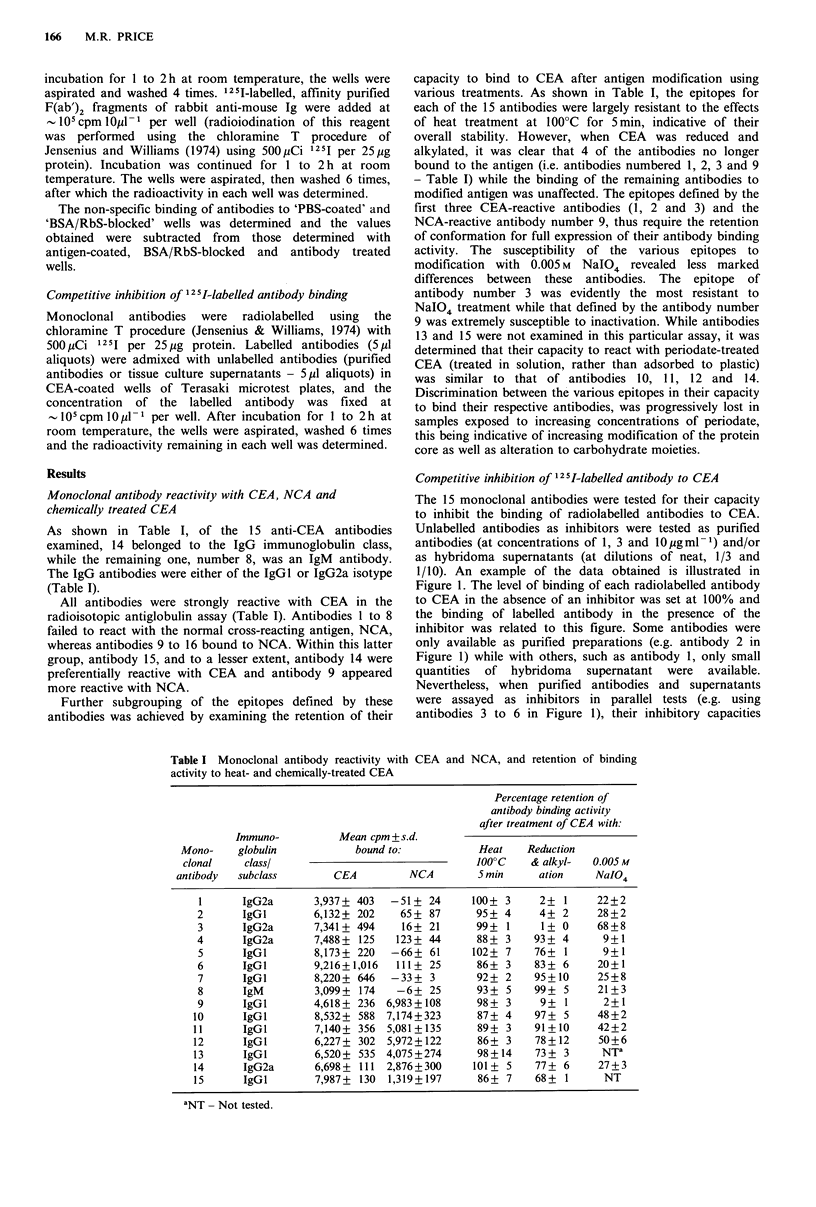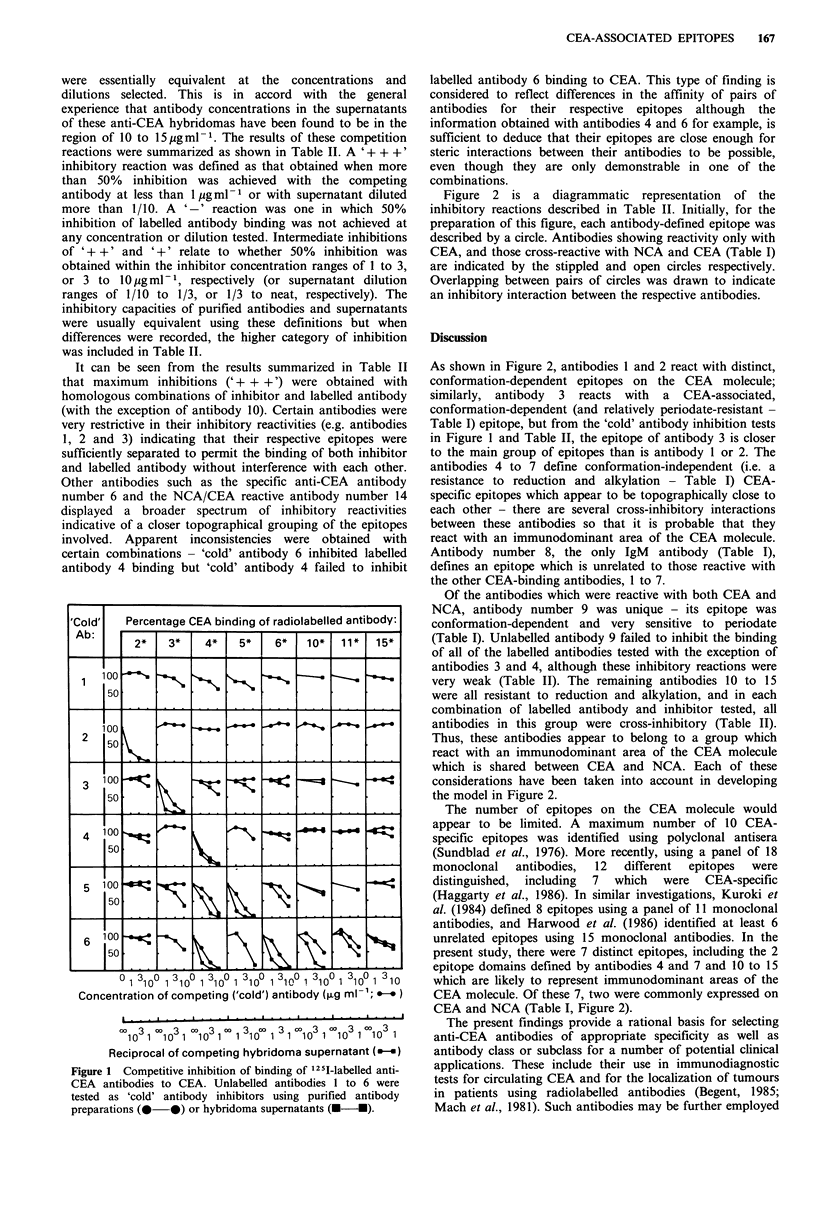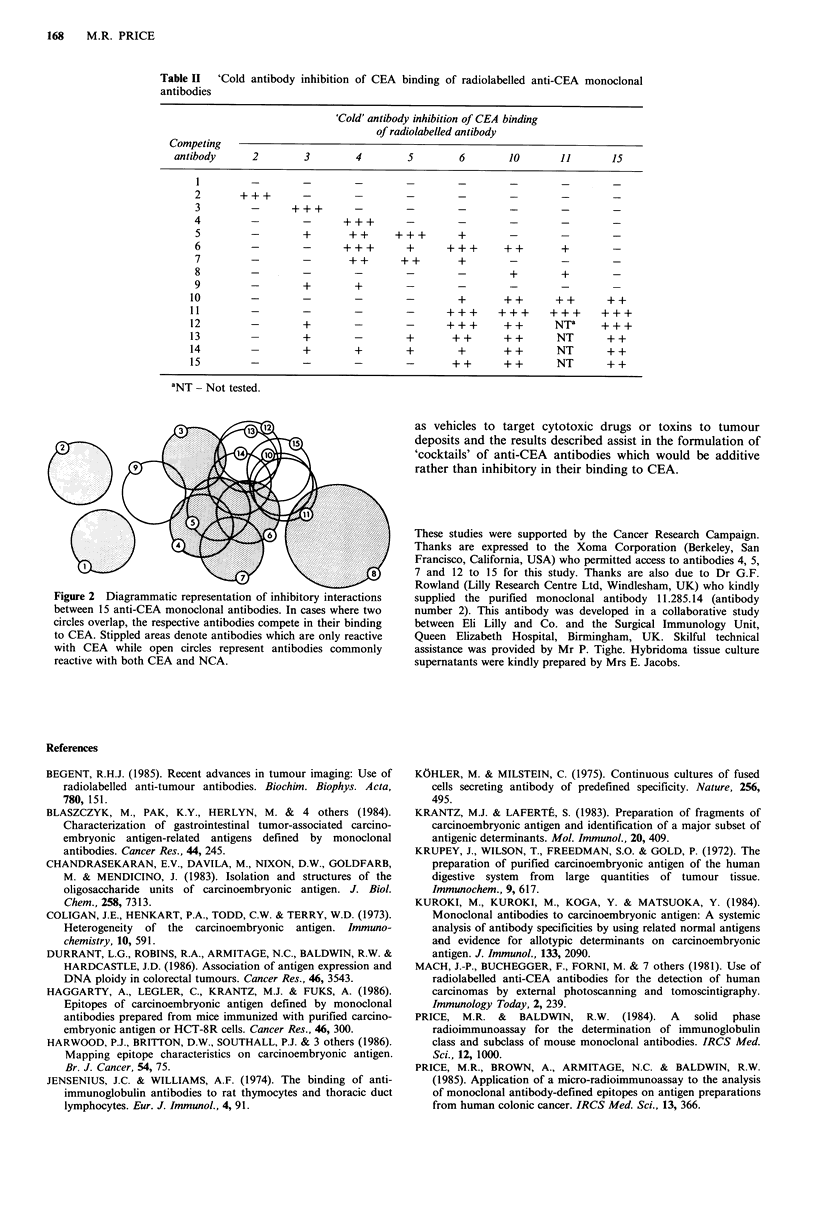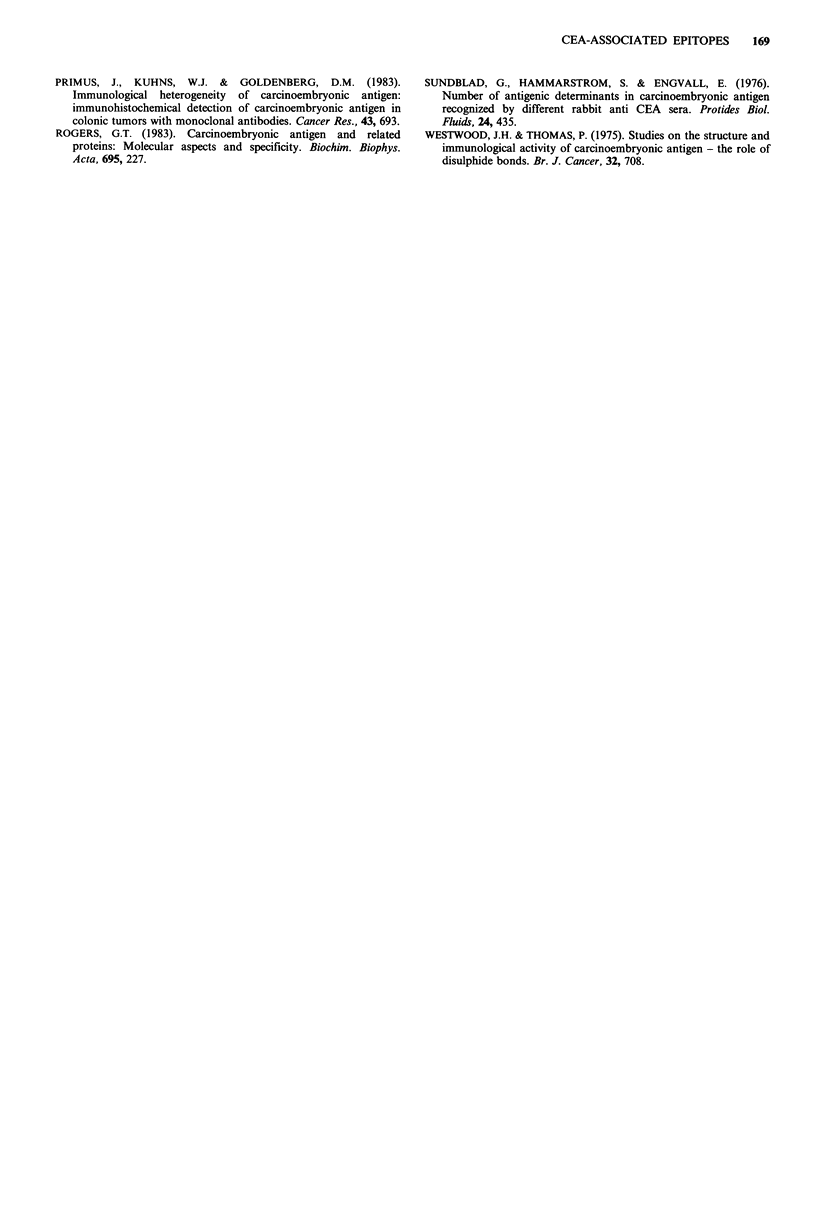# Epitopes of carcinoembryonic antigen (CEA) defined by monoclonal antibodies.

**DOI:** 10.1038/bjc.1988.34

**Published:** 1988-02

**Authors:** M. R. Price

**Affiliations:** Cancer Research Campaign Laboratories, University of Nottingham, University Park, UK.

## Abstract

Of 15 anti-CEA monoclonal antibodies, the first 8 were reactive only with CEA, while the remaining 7 antibodies reacted with epitopes commonly expressed on CEA and the normal cross-reacting antigen, NCA. Separate and distinct, conformation-dependent (i.e. susceptible to reduction and alkylation), CEA-associated epitopes were identified using antibodies 1, 2 and 3. Antibodies 4 to 7 defined a series of conformation-independent epitopes which were topographically closely related on the CEA molecule. Antibody number 8 reacted with a separate determinant found on CEA but not NCA, and this also was resistant to reduction and alkylation. Antibody number 9 defined an epitope which was commonly expressed on CEA and NCA. This epitope was conformation-dependent and was the most sensitive to NaIO4. The remaining antibodies, 10 to 15, which also reacted with CEA and NCA, defined an immunodominant region of these molecules since the 6 epitopes were clearly closely related, but not necessarily identical. The findings presented establish a rational basis for the selection of combinations of anti-CEA antibodies for diagnostic and therapeutic purposes.


*Br. J. Cancer* (1988), **57**, 165–169 © The Macmillan Press Ltd., 1988

### Epitopes of carcinoembryonic antigen (CEA) defined by monoclonal antibodies

M.R. Price

*Cancer Research Campaign Laboratories, University of Nottingham, University Park, Nottingham NG7 2RD, UK.*

**Summary** Of 15 anti-CEA monoclonal antibodies, the first 8 were reactive only with CEA, while the remaining 7 antibodies reacted with epitopes commonly expressed on CEA and the normal cross-reacting antigen, NCA. Separate and distinct, conformation-dependent (i.e. susceptible to reduction and alkylation), CEA-associated epitopes were identified using antibodies 1, 2 and 3. Antibodies 4 to 7 defined a series of conformation-independent epitopes which were topographically closely related on the CEA molecule. Antibody number 8 reacted with a separate determinant found on CEA but not NCA, and this also was resistant to reduction and alkylation.

Antibody number 9 defined an epitope which was commonly expressed on CEA and NCA. This epitope was conformation-dependent and was the most sensitive to $NaIO_4$. The remaining antibodies, 10 to 15, which also reacted with CEA and NCA, defined an immunodominant region of these molecules since the 6 epitopes were clearly closely related, but not necessarily identical.

The findings presented establish a rational basis for the selection of combinations of anti-CEA antibodies for diagnostic and therapeutic purposes.

Carcinoembryonic antigen (CEA) is a carbohydrate-rich glycoprotein, often expressed in tumours of the human gastro-intestinal tract, and which consists of a single polypeptide chain with about 40 N-linked oligosaccharide chains (Rogers, 1983; Chandrasekaran *et al.*, 1983). Purified CEA preparations exhibit microheterogeneity which has been attributed to variations in its associated oligosaccharides (Coligan *et al.*, 1973; Primus *et al.*, 1983) and frequently immunochemical procedures have been employed to define the structure of CEA. Monoclonal antibodies have proved to be particularly valuable in this respect and epitopes specifically expressed on CEA molecules have been clearly distinguished from other epitopes commonly found on CEA molecules and immunologically-related glycoproteins associated with certain normal tissues (Kuroki *et al.*, 1984; Blaszczyk *et al.*, 1984; Haggarty *et al.*, 1986).

In the present investigation, a panel of 15 anti-CEA monoclonal antibodies have been used to probe the epitope structure of CEA molecules and to establish a basis for the selection of reagents for antibody directed targeting to tumours of both radioisotopes for immunoscintigraphy and cytotoxic drugs for therapy.

### Materials and methods

### *Monoclonal antibodies*

Monoclonal antibodies 1 to 15 were prepared by conventional hybridoma technology by which the spleen cells from immunized BALB/c mice were fused with cells of the mouse myeloma, P3NS1 (Köhler & Milstein, 1975). Mice were immunized with purified CEA, colorectal tumour subcellular membranes or viable colonic tumour cells and antibodies were selected for their reactivity with CEA (Price *et al.*, 1985; Durrant *et al.*, 1986). All antibodies were the products of separate fusions with the exception of antibodies 6 and 7, and antibodies 12 and 13. The immunoglobulin class and subclass of antibodies was determined as previously described (Price & Baldwin, 1984). Monoclonal antibodies were purified from ascitic fluids or from hybridoma tissue culture supernatants by their binding to and elution from Sepharose-protein A (Pharmacia, Uppsala, Sweden).

Received 5 April 1987; and in revised form, 14 September 1987

### *Antigen preparations*

CEA was purified from hepatic metastases from primary colonic adenocarcinoma according to the method of Krupey *et al.*, (1972) and NCA was isolated from human spleen as previously described (Blaszczyk *et al.*, 1984; Price *et al.*, 1985). The purity of both antigen preparations was examined by sodium dodecyl sulphate polyacrylamide gel electrophoresis. CEA gave a single diffuse band migrating with an apparent molecular weight of 180 kD while NCA preparations showed a single band at 60 kD.

### *Chemical treatment of CEA*

CEA was reduced and alkylated with iodoacetamide according to Krantz and Laferté (1983). CEA ($0.25\,mg\,ml^{-1}$) was dissolved in 0.7 M Tris-HCl, pH 8.8, containing 0.2% SDS. After flushing the vial with nitrogen, 15 mM dithiothreitol was added, and it was sealed and heated at 100°C for 5 min. After cooling to room temperature, 50 mM iodoacetamide was added and the reaction mixture was kept in the dark for 30 min. Reagents were then removed by passage through a column of Sephadex G25 (Pharmacia, Uppsala, Sweden) equilibrated with PBS, pH 7.3. CEA preparations were also treated with $NaIO_4$ solutions (Westwood & Thomas, 1975). CEA was dried onto the surface of wells of Terasaki microtest plates (well capacity – 10 $\mu$l) as described in the following section. Adsorbed CEA was then treated with 0.005 M $NaIO_4$ for 20 h at room temperature in the dark. Reagents were removed by washing the microtest plates with PBS.

### *Radioisotopic antiglobulin test*

Antigen preparations ($50\,\mu g\,ml^{-1}$ in PBS + 0.02% $NaN_3$) were added to 60-well Terasaki microtest plates (well capacity – 10 $\mu$l; Labtech Division, Miles Laboratories, Naperville, IL, USA) at 10 $\mu$l per well. Plates containing CEA or NCA were air dried by overnight incubation at 37°C. The wells were washed 4 times with a washing buffer consisting of PBS + 0.1% bovine serum albumin (BSA) + 0.1% rabbit serum (RbS) + 0.02% $NaN_3$. During the final wash cycle, the wells were incubated with the washing buffer for at least 30 min to complete the blocking of non-specific binding adsorption sites. Hybridoma supernatants or purified monoclonal antibodies (at the saturating concentration of $1\,\mu g\,ml^{-1}$ in washing buffer), or washing buffer alone in negative controls, were added at 10 $\mu$l per well. After incubation for 1 to 2h at room temperature, the wells were aspirated and washed 4 times. $^{125}$I-labelled, affinity purified $F(ab')_2$ fragments of rabbit anti-mouse Ig were added at $\sim 10^5$ cpm $10\mu l^{-1}$ per well (radioiodination of this reagent was performed using the chloramine T procedure of Jensenius and Williams (1974) using $500\mu$Ci $^{125}$I per $25\mu g$ protein). Incubation was continued for 1 to 2h at room temperature. The wells were aspirated, then washed 6 times, after which the radioactivity in each well was determined.

The non-specific binding of antibodies to 'PBS-coated' and 'BSA/RbS-blocked' wells was determined and the values obtained were subtracted from those determined with antigen-coated, BSA/RbS-blocked and antibody treated wells.

### *Competitive inhibition of $^{125}$I-labelled antibody binding*

Monoclonal antibodies were radiolabelled using the chloramine T procedure (Jensenius & Williams, 1974) with $500\mu$Ci $^{125}$I per $25\mu g$ protein. Labelled antibodies ($5\mu l$ aliquots) were admixed with unlabelled antibodies (purified antibodies or tissue culture supernatants – $5\mu l$ aliquots) in CEA-coated wells of Terasaki microtest plates, and the concentration of the labelled antibody was fixed at $\sim 10^5$ cpm $10\mu l^{-1}$ per well. After incubation for 1 to 2h at room temperature, the wells were aspirated, washed 6 times and the radioactivity remaining in each well was determined.

### Results

### *Monoclonal antibody reactivity with CEA, NCA and chemically treated CEA*

As shown in Table I, of the 15 anti-CEA antibodies examined, 14 belonged to the IgG immunoglobulin class, while the remaining one, number 8, was an IgM antibody. The IgG antibodies were either of the IgG1 or IgG2a isotype (Table I).

All antibodies were strongly reactive with CEA in the radioisotopic antiglobulin assay (Table I). Antibodies 1 to 8 failed to react with the normal cross-reacting antigen, NCA, whereas antibodies 9 to 16 bound to NCA. Within this latter group, antibody 15, and to a lesser extent, antibody 14 were preferentially reactive with CEA and antibody 9 appeared more reactive with NCA.

Further subgrouping of the epitopes defined by these antibodies was achieved by examining the retention of their capacity to bind to CEA after antigen modification using various treatments. As shown in Table I, the epitopes for each of the 15 antibodies were largely resistant to the effects of heat treatment at 100°C for 5min, indicative of their overall stability. However, when CEA was reduced and alkylated, it was clear that 4 of the antibodies no longer bound to the antigen (i.e. antibodies numbered 1, 2, 3 and 9 – Table I) while the binding of the remaining antibodies to modified antigen was unaffected. The epitopes defined by the first three CEA-reactive antibodies (1, 2 and 3) and the NCA-reactive antibody number 9, thus require the retention of conformation for full expression of their antibody binding activity. The susceptibility of the various epitopes to modification with 0.005 M $NaIO_4$ revealed less marked differences between these antibodies. The epitope of antibody number 3 was evidently the most resistant to $NaIO_4$ treatment while that defined by the antibody number 9 was extremely susceptible to inactivation. While antibodies 13 and 15 were not examined in this particular assay, it was determined that their capacity to react with periodate-treated CEA (treated in solution, rather than adsorbed to plastic) was similar to that of antibodies 10, 11, 12 and 14. Discrimination between the various epitopes in their capacity to bind their respective antibodies, was progressively lost in samples exposed to increasing concentrations of periodate, this being indicative of increasing modification of the protein core as well as alteration to carbohydrate moieties.

### *Competitive inhibition of $^{125}$I-labelled antibody to CEA*

The 15 monoclonal antibodies were tested for their capacity to inhibit the binding of radiolabelled antibodies to CEA. Unlabelled antibodies as inhibitors were tested as purified antibodies (at concentrations of 1, 3 and $10\mu g\,ml^{-1}$) and/or as hybridoma supernatants (at dilutions of neat, 1/3 and 1/10). An example of the data obtained is illustrated in Figure 1. The level of binding of each radiolabelled antibody to CEA in the absence of an inhibitor was set at 100% and the binding of labelled antibody in the presence of the inhibitor was related to this figure. Some antibodies were only available as purified preparations (e.g. antibody 2 in Figure 1) while with others, such as antibody 1, only small quantities of hybridoma supernatant were available. Nevertheless, when purified antibodies and supernatants were assayed as inhibitors in parallel tests (e.g. using antibodies 3 to 6 in Figure 1), their inhibitory capacities

**Table I** Monoclonal antibody reactivity with CEA and NCA, and retention of binding activity to heat- and chemically-treated CEA

| Monoclonal antibody | Immunoglobulin class/subclass | Mean cpm ± s.d. bound to: CEA | NCA | Percentage retention of antibody binding activity after treatment of CEA with: Heat 100°C 5 min | Reduction & alkylation | 0.005 M $NaIO_4$ |
|---|---|---|---|---|---|---|
| 1 | IgG2a | 3,937 ± 403 | −51 ± 24 | 100 ± 3 | 2 ± 1 | 22 ± 2 |
| 2 | IgG1 | 6,132 ± 202 | 65 ± 87 | 95 ± 4 | 4 ± 2 | 28 ± 2 |
| 3 | IgG2a | 7,341 ± 494 | 16 ± 21 | 99 ± 1 | 1 ± 0 | 68 ± 8 |
| 4 | IgG2a | 7,488 ± 125 | 123 ± 44 | 88 ± 3 | 93 ± 4 | 9 ± 1 |
| 5 | IgG1 | 8,173 ± 220 | −66 ± 61 | 102 ± 7 | 76 ± 1 | 9 ± 1 |
| 6 | IgG1 | 9,216 ± 1,016 | 111 ± 25 | 86 ± 3 | 83 ± 6 | 20 ± 1 |
| 7 | IgG1 | 8,220 ± 646 | −33 ± 3 | 92 ± 2 | 95 ± 10 | 25 ± 8 |
| 8 | IgM | 3,099 ± 174 | −6 ± 25 | 93 ± 5 | 99 ± 5 | 21 ± 3 |
| 9 | IgG1 | 4,618 ± 236 | 6,983 ± 108 | 98 ± 3 | 9 ± 1 | 2 ± 1 |
| 10 | IgG1 | 8,532 ± 588 | 7,174 ± 323 | 87 ± 4 | 97 ± 5 | 48 ± 2 |
| 11 | IgG1 | 7,140 ± 356 | 5,081 ± 135 | 89 ± 3 | 91 ± 10 | 42 ± 2 |
| 12 | IgG1 | 6,227 ± 302 | 5,972 ± 122 | 86 ± 3 | 78 ± 12 | 50 ± 6 |
| 13 | IgG1 | 6,520 ± 535 | 4,075 ± 274 | 98 ± 14 | 73 ± 3 | NT[a] |
| 14 | IgG2a | 6,698 ± 111 | 2,876 ± 300 | 101 ± 5 | 77 ± 6 | 27 ± 3 |
| 15 | IgG1 | 7,987 ± 130 | 1,319 ± 197 | 86 ± 7 | 68 ± 1 | NT |

[a]NT – Not tested.

were essentially equivalent at the concentrations and dilutions selected. This is in accord with the general experience that antibody concentrations in the supernatants of these anti-CEA hybridomas have been found to be in the region of 10 to 15 $\mu g\,ml^{-1}$. The results of these competition reactions were summarized as shown in Table II. A '+ + +' inhibitory reaction was defined as that obtained when more than 50% inhibition was achieved with the competing antibody at less than 1 $\mu g\,ml^{-1}$ or with supernatant diluted more than 1/10. A '−' reaction was one in which 50% inhibition of labelled antibody binding was not achieved at any concentration or dilution tested. Intermediate inhibitions of '+ +' and '+' relate to whether 50% inhibition was obtained within the inhibitor concentration ranges of 1 to 3, or 3 to 10 $\mu g\,ml^{-1}$, respectively (or supernatant dilution ranges of 1/10 to 1/3, or 1/3 to neat, respectively). The inhibitory capacities of purified antibodies and supernatants were usually equivalent using these definitions but when differences were recorded, the higher category of inhibition was included in Table II.

It can be seen from the results summarized in Table II that maximum inhibitions ('+ + +') were obtained with homologous combinations of inhibitor and labelled antibody (with the exception of antibody 10). Certain antibodies were very restrictive in their inhibitory reactivities (e.g. antibodies 1, 2 and 3) indicating that their respective epitopes were sufficiently separated to permit the binding of both inhibitor and labelled antibody without interference with each other. Other antibodies such as the specific anti-CEA antibody number 6 and the NCA/CEA reactive antibody number 14 displayed a broader spectrum of inhibitory reactivities indicative of a closer topographical grouping of the epitopes involved. Apparent inconsistencies were obtained with certain combinations – 'cold' antibody 6 inhibited labelled antibody 4 binding but 'cold' antibody 4 failed to inhibit

**Figure 1** Competitive inhibition of binding of $^{125}$I-labelled anti-CEA antibodies to CEA. Unlabelled antibodies 1 to 6 were tested as 'cold' antibody inhibitors using purified antibody preparations (●——●) or hybridoma supernatants (■——■).

labelled antibody 6 binding to CEA. This type of finding is considered to reflect differences in the affinity of pairs of antibodies for their respective epitopes although the information obtained with antibodies 4 and 6 for example, is sufficient to deduce that their epitopes are close enough for steric interactions between their antibodies to be possible, even though they are only demonstrable in one of the combinations.

Figure 2 is a diagrammatic representation of the inhibitory reactions described in Table II. Initially, for the preparation of this figure, each antibody-defined epitope was described by a circle. Antibodies showing reactivity only with CEA, and those cross-reactive with NCA and CEA (Table I) are indicated by the stippled and open circles respectively. Overlapping between pairs of circles was drawn to indicate an inhibitory interaction between the respective antibodies.

### Discussion

As shown in Figure 2, antibodies 1 and 2 react with distinct, conformation-dependent epitopes on the CEA molecule; similarly, antibody 3 reacts with a CEA-associated, conformation-dependent (and relatively periodate-resistant – Table I) epitope, but from the 'cold' antibody inhibition tests in Figure 1 and Table II, the epitope of antibody 3 is closer to the main group of epitopes than is antibody 1 or 2. The antibodies 4 to 7 define conformation-independent (i.e. a resistance to reduction and alkylation – Table I) CEA-specific epitopes which appear to be topographically close to each other – there are several cross-inhibitory interactions between these antibodies so that it is probable that they react with an immunodominant area of the CEA molecule. Antibody number 8, the only IgM antibody (Table I), defines an epitope which is unrelated to those reactive with the other CEA-binding antibodies, 1 to 7.

Of the antibodies which were reactive with both CEA and NCA, antibody number 9 was unique – its epitope was conformation-dependent and very sensitive to periodate (Table I). Unlabelled antibody 9 failed to inhibit the binding of all of the labelled antibodies tested with the exception of antibodies 3 and 4, although these inhibitory reactions were very weak (Table II). The remaining antibodies 10 to 15 were all resistant to reduction and alkylation, and in each combination of labelled antibody and inhibitor tested, all antibodies in this group were cross-inhibitory (Table II). Thus, these antibodies appear to belong to a group which react with an immunodominant area of the CEA molecule which is shared between CEA and NCA. Each of these considerations have been taken into account in developing the model in Figure 2.

The number of epitopes on the CEA molecule would appear to be limited. A maximum number of 10 CEA-specific epitopes was identified using polyclonal antisera (Sundblad *et al.*, 1976). More recently, using a panel of 18 monoclonal antibodies, 12 different epitopes were distinguished, including 7 which were CEA-specific (Haggarty *et al.*, 1986). In similar investigations, Kuroki *et al.* (1984) defined 8 epitopes using a panel of 11 monoclonal antibodies, and Harwood *et al.* (1986) identified at least 6 unrelated epitopes using 15 monoclonal antibodies. In the present study, there were 7 distinct epitopes, including the 2 epitope domains defined by antibodies 4 and 7 and 10 to 15 which are likely to represent immunodominant areas of the CEA molecule. Of these 7, two were commonly expressed on CEA and NCA (Table I, Figure 2).

The present findings provide a rational basis for selecting anti-CEA antibodies of appropriate specificity as well as antibody class or subclass for a number of potential clinical applications. These include their use in immunodiagnostic tests for circulating CEA and for the localization of tumours in patients using radiolabelled antibodies (Begent, 1985; Mach *et al.*, 1981). Such antibodies may be further employed

**Table II** 'Cold antibody inhibition of CEA binding of radiolabelled anti-CEA monoclonal antibodies

| *Competing antibody* | *'Cold' antibody inhibition of CEA binding of radiolabelled antibody* 2 | 3 | 4 | 5 | 6 | 10 | 11 | 15 |
|---|---|---|---|---|---|---|---|---|
| 1 | − | − | − | − | − | − | − | − |
| 2 | +++ | − | − | − | − | − | − | − |
| 3 | − | +++ | − | − | − | − | − | − |
| 4 | − | − | +++ | − | − | − | − | − |
| 5 | − | + | ++ | +++ | + | − | − | − |
| 6 | − | − | +++ | + | +++ | ++ | + | − |
| 7 | − | − | ++ | ++ | + | − | − | − |
| 8 | − | − | − | − | − | + | + | − |
| 9 | − | + | + | − | − | − | − | − |
| 10 | − | − | − | − | + | ++ | ++ | ++ |
| 11 | − | − | − | − | +++ | +++ | +++ | +++ |
| 12 | − | + | − | − | +++ | ++ | NT[a] | +++ |
| 13 | − | + | − | + | ++ | ++ | NT | ++ |
| 14 | − | + | + | + | + | ++ | NT | ++ |
| 15 | − | − | − | − | ++ | ++ | NT | ++ |

[a]NT - Not tested.

**Figure 2** Diagrammatic representation of inhibitory interactions between 15 anti-CEA monoclonal antibodies. In cases where two circles overlap, the respective antibodies compete in their binding to CEA. Stippled areas denote antibodies which are only reactive with CEA while open circles represent antibodies commonly reactive with both CEA and NCA.

as vehicles to target cytotoxic drugs or toxins to tumour deposits and the results described assist in the formulation of 'cocktails' of anti-CEA antibodies which would be additive rather than inhibitory in their binding to CEA.

These studies were supported by the Cancer Research Campaign. Thanks are expressed to the Xoma Corporation (Berkeley, San Francisco, California, USA) who permitted access to antibodies 4, 5, 7 and 12 to 15 for this study. Thanks are also due to Dr G.F. Rowland (Lilly Research Centre Ltd, Windlesham, UK) who kindly supplied the purified monoclonal antibody 11.285.14 (antibody number 2). This antibody was developed in a collaborative study between Eli Lilly and Co. and the Surgical Immunology Unit, Queen Elizabeth Hospital, Birmingham, UK. Skilful technical assistance was provided by Mr P. Tighe. Hybridoma tissue culture supernatants were kindly prepared by Mrs E. Jacobs.

### References

BEGENT, R.H.J. (1985). Recent advances in tumour imaging: Use of radiolabelled anti-tumour antibodies. *Biochim. Biophys. Acta,* **780,** 151.

BLASZCZYK, M., PAK, K.Y., HERLYN, M. & 4 others (1984). Characterization of gastrointestinal tumor-associated carcinoembryonic antigen-related antigens defined by monoclonal antibodies. *Cancer Res.,* **44,** 245.

CHANDRASEKARAN, E.V., DAVILA, M., NIXON, D.W., GOLDFARB, M. & MENDICINO, J. (1983). Isolation and structures of the oligosaccharide units of carcinoembryonic antigen. *J. Biol. Chem.,* **258,** 7313.

COLIGAN, J.E., HENKART, P.A., TODD, C.W. & TERRY, W.D. (1973). Heterogeneity of the carcinoembryonic antigen. *Immunochemistry,* **10,** 591.

DURRANT, L.G., ROBINS, R.A., ARMITAGE, N.C., BALDWIN, R.W. & HARDCASTLE, J.D. (1986). Association of antigen expression and DNA ploidy in colorectal tumours. *Cancer Res.,* **46,** 3543.

HAGGARTY, A., LEGLER, C., KRANTZ, M.J. & FUKS, A. (1986). Epitopes of carcinoembryonic antigen defined by monoclonal antibodies prepared from mice immunized with purified carcinoembryonic antigen or HCT-8R cells. *Cancer Res.,* **46,** 300.

HARWOOD, P.J., BRITTON, D.W., SOUTHALL, P.J. & 3 others (1986). Mapping epitope characteristics on carcinoembryonic antigen. *Br. J. Cancer,* **54,** 75.

JENSENIUS, J.C. & WILLIAMS, A.F. (1974). The binding of anti-immunoglobulin antibodies to rat thymocytes and thoracic duct lymphocytes. *Eur. J. Immunol.,* **4,** 91.

KÖHLER, M. & MILSTEIN, C. (1975). Continuous cultures of fused cells secreting antibody of predefined specificity. *Nature,* **256,** 495.

KRANTZ, M.J. & LAFERTÉ, S. (1983). Preparation of fragments of carcinoembryonic antigen and identification of a major subset of antigenic determinants. *Mol. Immunol.,* **20,** 409.

KRUPEY, J., WILSON, T., FREEDMAN, S.O. & GOLD, P. (1972). The preparation of purified carcinoembryonic antigen of the human digestive system from large quantities of tumour tissue. *Immunochem.,* **9,** 617.

KUROKI, M., KUROKI, M., KOGA, Y. & MATSUOKA, Y. (1984). Monoclonal antibodies to carcinoembryonic antigen: A systemic analysis of antibody specificities by using related normal antigens and evidence for allotypic determinants on carcinoembryonic antigen. *J. Immunol.,* **133,** 2090.

MACH, J.-P., BUCHEGGER, F., FORNI, M. & 7 others (1981). Use of radiolabelled anti-CEA antibodies for the detection of human carcinomas by external photoscanning and tomoscintigraphy. *Immunology Today,* **2,** 239.

PRICE, M.R. & BALDWIN, R.W. (1984). A solid phase radioimmunoassay for the determination of immunoglobulin class and subclass of mouse monoclonal antibodies. *IRCS Med. Sci.,* **12,** 1000.

PRICE, M.R., BROWN, A., ARMITAGE, N.C. & BALDWIN, R.W. (1985). Application of a micro-radioimmunoassay to the analysis of monoclonal antibody-defined epitopes on antigen preparations from human colonic cancer. *IRCS Med. Sci.,* **13,** 366.

PRIMUS, J., KUHNS, W.J. & GOLDENBERG, D.M. (1983). Immunological heterogeneity of carcinoembryonic antigen: immunohistochemical detection of carcinoembryonic antigen in colonic tumors with monoclonal antibodies. *Cancer Res.*, **43**, 693.

ROGERS, G.T. (1983). Carcinoembryonic antigen and related proteins: Molecular aspects and specificity. *Biochim. Biophys. Acta*, **695**, 227.

SUNDBLAD, G., HAMMARSTROM, S. & ENGVALL, E. (1976). Number of antigenic determinants in carcinoembryonic antigen recognized by different rabbit anti CEA sera. *Protides Biol. Fluids*, **24**, 435.

WESTWOOD, J.H. & THOMAS, P. (1975). Studies on the structure and immunological activity of carcinoembryonic antigen – the role of disulphide bonds. *Br. J. Cancer*, **32**, 708.